# The role of IBV PL1pro in virus replication and suppression of host innate immune responses

**DOI:** 10.1186/s12917-023-03839-2

**Published:** 2023-12-13

**Authors:** Weirong Liu, Ge Mu, Yiquan Jia, Mengting Yu, Songbai Zhang, Zhen Wang, Shouguo Fang

**Affiliations:** 1 Yangtze University Health Science Center, Jingzhou, Hubei Province China; 2 College of Agriculture, Yangtze University, No.88, Jingmi Road, Jingzhou, Hubei Province 434025 China; 3 MARA Key Laboratory of Sustainable Crop Production in the Middle Reaches of the Yangtze River (Co-construction by Ministry and Province), College of Agriculture, Yangtze University, Jingzhou, Hubei Province China

**Keywords:** Infectious Bronchitis virus, Papain-like protease, Virus replication, Interferon, Immune response

## Abstract

**Background:**

Coronavirus papain-like proteases (PLpros) play a crucial role in virus replication and the evasion of the host immune response. Infectious bronchitis virus (IBV) encodes a proteolytically defective remnant of PL1pro and an active PL2pro. However, the function of PL1pro in IBV remains largely unknown. This study aims to explore the effect of PL1pro on virus replication and underlying mechanisms.

**Results:**

The recombinant viruses rIBV-ΔPL1pro and rIBV-ΔPL1pro-N were obtained using reverse genetic techniques through the deletion of the IBV PL1pro domain and the N-terminal conserved sequence of PL1pro (PL1pro-N). We observed significantly lower replication of rIBV-ΔPL1pro and rIBV-ΔPL1pro-N than wild-type IBV. Further investigation revealed that the lack of PL1pro-N in IBV decreased virus resistance to interferon (IFN) while also inducing host immune response by enhancing the production of IFN-β and activating the downstream STAT1 signaling pathway of IFNs. In addition, the overexpression of PL1pro-N significantly suppressed type I IFN response by down-regulating the expressions of genes in the IFN pathway.

**Conclusions:**

Our data demonstrated that IBV PL1pro plays a crucial role in IBV replication and the suppression of host innate immune responses, suggesting that IBV PL1pro could serve as a promising molecular target for antiviral therapy.

**Supplementary Information:**

The online version contains supplementary material available at 10.1186/s12917-023-03839-2.

## Background

Coronaviruses are significant pathogens causing respiratory diseases in humans and many animal species [1]. Avian infectious bronchitis virus (IBV) is classified under the genus *Gammacoronavirus*. Although it has been recognized since the 1930s, IBV remains a primary cause of disease and economic losses in the poultry industry [2]. IBV is an enveloped virus with a single-stranded positive-sense RNA genome of approximately 27 kb [3]. The 3’-end of the viral genome encodes structural and accessory proteins, the 5’-end, which accounts for about two-thirds of the viral genome, encodes two overlapping polyproteins, which are proteolytically cleaved into 15 functional non-structural proteins (nsp2–nsp16) by virus-encoded proteinases, one or two papain-like proteases (PLpros), termed PL1pro and PL2pro and 3 C-like cysteine protease (3CLpro) [4–6].

Papain-like protease domains in the nsp3 of coronaviruses are essential for releasing nsp1, nsp2, and nsp3 from the N-terminal region of polyproteins. Besides their function in the viral replicase polyproteins processing, PLpros also play a crucial role in antagonizing interferon (IFN) response through their deubiquitinase (DUB) activity [7–10]. While the PL2pro domain is conserved across all coronaviruses, the functional PL1pro domain follows the hypervariable region (HVR) in nsp3 is exclusive to *Alphacoronavirus* and clade A of *Betacoronavirus* [11]. Only one structure of a PL1pro domain from the *Alphacoronavirus* transmissible gastroenteritis virus (TGEV) has been determined [12]. The structure of TGEV PL1pro resembles an extended right hand with thumb, palm, and finger subdomains. The finger subdomain contains a catalytic triad, Cys32 − His183 − Asp196, and a zinc finger region composed of four cysteine residues (Cys103, Cys105, Cys131, and Cys134), which are responsible for processing the viral polyproteins. The function of PL1pro remains poorly understood, primarily due to its absence in certain coronaviruses. PL1pro plays a crucial role in viral replication and host immune evasion in viruses that encode it. The PL1pro of Human coronavirus NL63 directly interacts with the host E3 ubiquitin ligase RCHY1, thereby increasing the stability of the latter and augmenting RCHY1-mediated degradation of p53 to inhibit the p53-mediated production of type I IFN [13]. TGEV PL1pro exhibits IFN antagonist activity dependent on the intact catalytic triad (C32, H183, and D196) and interferes with RIG-1- and STING-mediated signaling through DUB activity [12]. Porcine epidemic diarrhea virus (PEDV) PL1pro negatively regulates the production of type 1 IFN by interacting with host cell poly(C) binding protein 2 (PCBP2) and promotes virus replication [14]. Given the crucial function of PLpros in processing viral replicase polyproteins and their capability to counteract host immune responses, they have been identified as key molecular targets for antiviral treatments [15, 16].

The *Gammacoronavirus* IBV encodes a proteolytically defective remnant of PL1pro and an active PL2pro, currently known as PLpro. The PL1pro in IBV is incomplete due to the absence of the zinc-finger motif and the catalytic triad residues [17]. Why has PL1pro, an enzyme with no enzymatic activity, been preserved during the long-term evolution of IBV? Are there undiscovered biological functions associated with it? This study explored the effect of IBV PL1pro on virus replication and the underlying mechanisms. We discovered that IBV PL1pro plays a crucial role in virus replication, as its absence significantly impeded IBV replication in vitro. Additionally, we investigated the potential role of IBV PL1pro in the host’s antiviral response following IBV infection. Our findings revealed that the N-terminal region of IBV PL1pro negatively regulates the IFN signaling pathway.

## Results

### Multiple sequence alignment of PL1pro from different IBV isolates

**Fig. 1:**
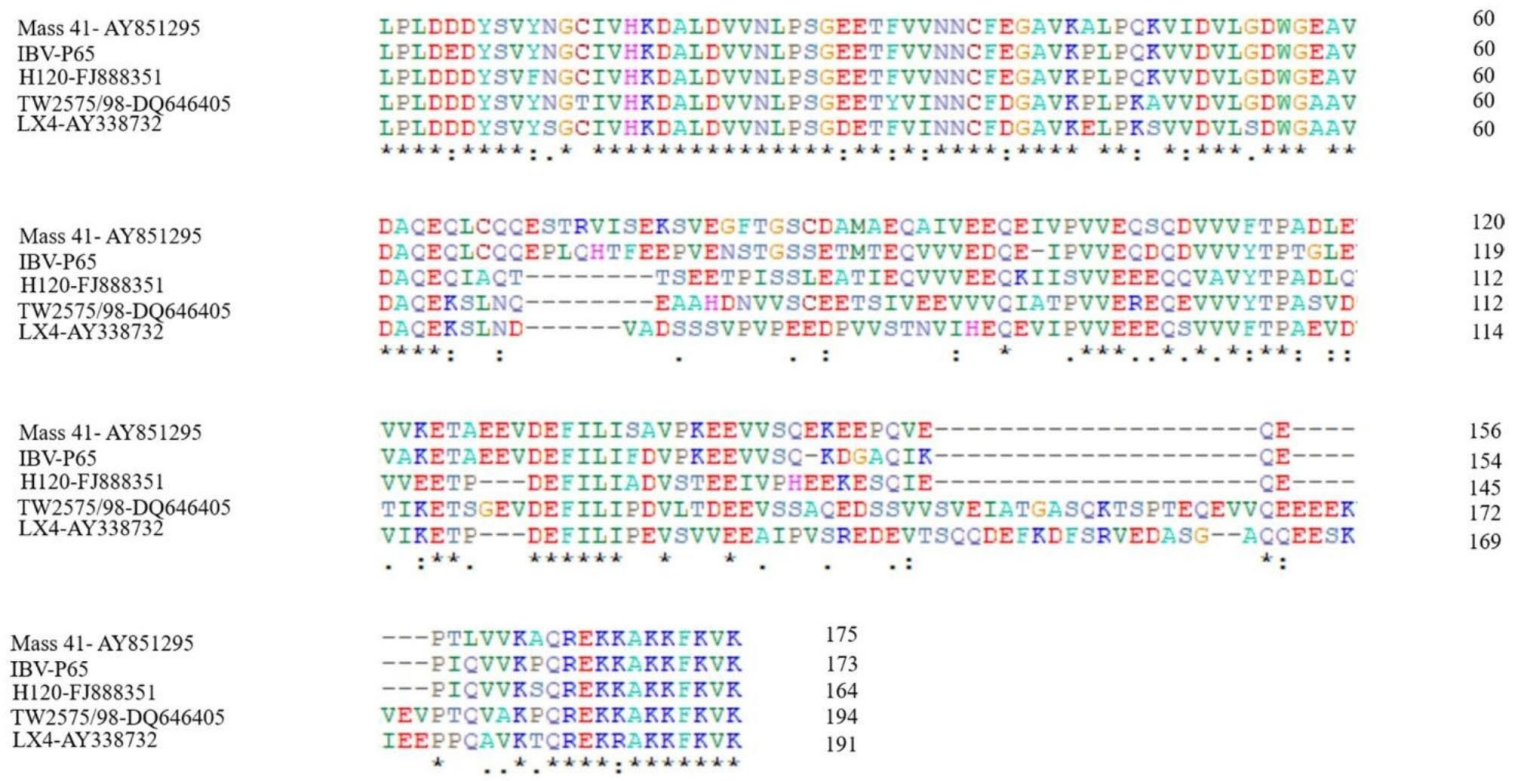
Multiple sequence alignments of PL1pro from different IBV isolates

This study utilized the IBV-Beaudette strain (IBV-P65) to investigate the involvement of PL1pro in IBV replication and the underlying mechanisms. PL1pro of IBV-P65 is located between two conserved functional domains: the Ac domain (Glu-rich acidic domain) and the macrodomain (also known as “X domain”), with a length of 519 nucleotides (3022–3540 nt), which encode 173 amino acids. Sequence alignment of PL1pro from different IBV isolates (IBV-P65, H120-FJ888351, LX4-AY338732, TW2575/98-DQ646405, Mass 41- AY851295) showed a high level of conservation in the N-terminal region (PL1pro-N) with 64 amino acid residues and in the C-terminal region (PL1pro-C) comprised of 12 amino acid residues (homology 92%–100%). However, the region between these two has significant variability in the amino acid residues (Fig. 1).

### Rescue of the infectious viruses rIBV, rIBV-ΔPL1pro, and rIBV-ΔPL1pro-N

**Fig. 2:**
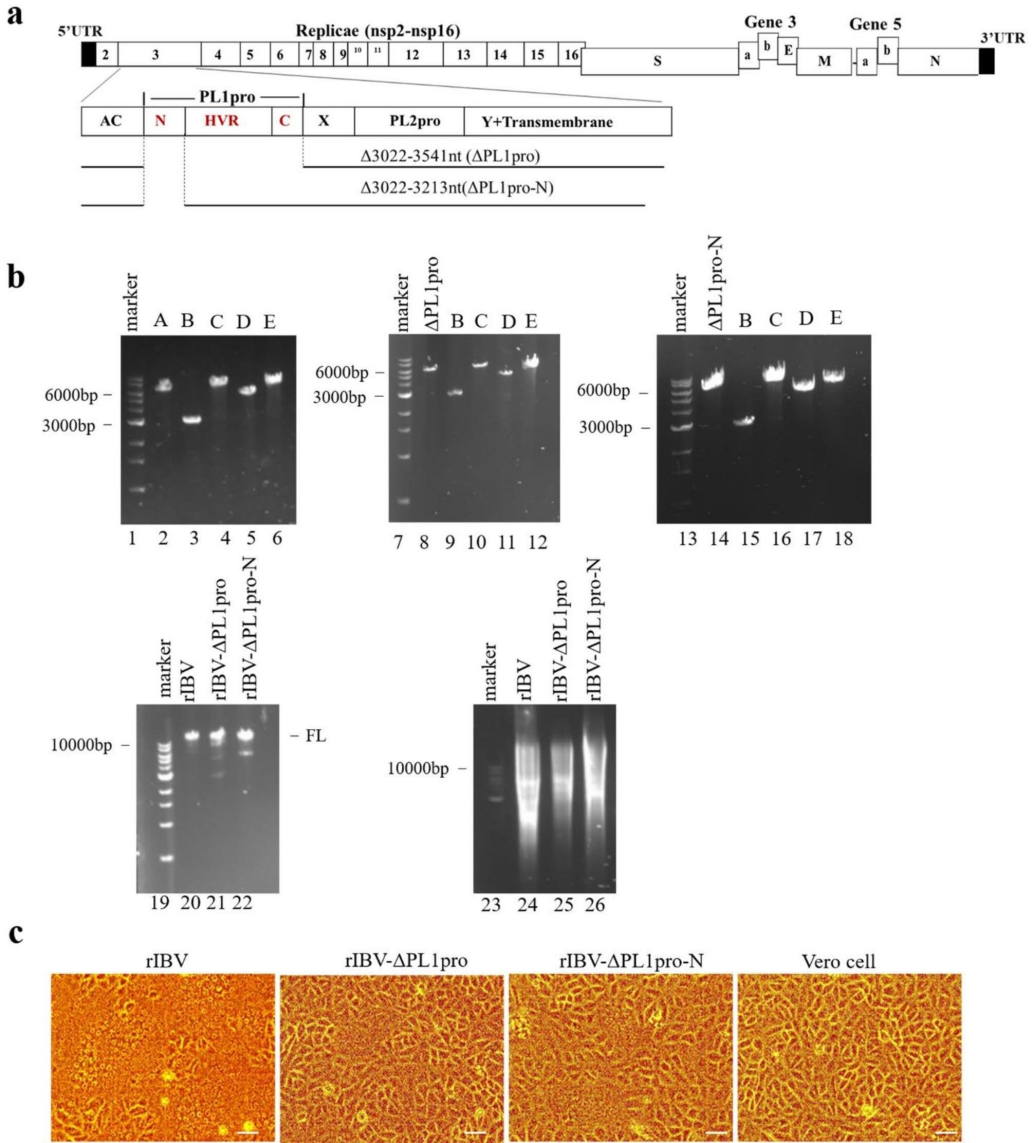
Recovery of rIBV, rIBV-ΔPL1pro, and rIBV-ΔPL1pro-N from cells electroporated with in vitro-synthesized full-length transcripts. (**a**) A schematic of the genome in nsp3 of rIBV, rIBV-ΔPL1pro, and rIBV-ΔPL1pro-N. (**b**) In vitro transcription of the full-length transcripts of rIBV, rIBV-ΔPL1pro, and rIBV-ΔPL1pro-N. Lanes 2–6,8–12,14–18: The five cDNA fragments covering IBV sequences from plasmids pKTO-IBV-A (pKTO-IBV-ΔPL1pro, pKTO-IBV-ΔPL1pro-N), pGEM-IBV-B, pXL-IBV-C, pGEM-IBV-D, and pGEM-IBV-E, respectively; Lanes 20–22: In vitro assembly of the five fragments into a full-length cDNA of rIBV, rIBV-ΔPL1pro, and rIBV-ΔPL1pro-N; Lanes 24–26: Generation of the full-length in vitro transcripts of rIBV, rIBV-ΔPL1pro, and rIBV-ΔPL1pro-N; Lanes 1, 7, 13, 19 and 23: DNA markers. The full-length gels are in Additional file 1: Supplemental Fig. 2. (**c**) CPE in Vero cells electroporated with in vitro-synthesized transcripts derived from the assembled full-length clones of rIBV, rIBV-ΔPL1pro, and rIBV-ΔPL1pro-N. Scale bars: 50 μm

To construct the full-length cDNA clones of rIBV, rIBV-ΔPL1pro, and rIBV-ΔPL1pro-N, we obtained five fragments (A to E) spanning the entire IBV genome via RT-PCR. These fragments were derived from Vero cells infected with the IBV Beaudette strain (p65) [18]. These five fragments were then ligated to produce the full-length IBV cDNA. In the latter two clones, the sequences of the IBV cDNA covering the entire PL1pro domain (3022–3540 nt) or the N-terminal region of PL1pro (3022–3213 nt) were eliminated, respectively (Fig. 2a). RNA transcripts from the three full-length cDNA clones were co-transfected with the N transcripts into Vero cells, an African green monkey kidney epithelial cell line, using electroporation. At 48 h post-electroporation, a typical cytopathic effect (CPE) of the Vero cell-adapted IBV, the formation of giant syncytial cells, was observed in cells transfected with transcripts from cDNA clones of rIBV, rIBV-ΔPL1pro, and rIBV-ΔPL1pro -N (Fig. 2b-c).

RT-PCR analysis of IBV subgenomic mRNA 2 was conducted to confirm the virus replication. The recovery of rIBV-ΔPL1pro and rIBV-ΔPL1pro-N from the in vitro-synthesized full-length transcripts was confirmed by sequencing the RT-PCR fragments that covered the deleted regions (Additional file 1: Supplemental Fig. 1).

### The absence of PL1pro or the N-terminal region of PL1pro weakens virus replication

**Fig. 3:**
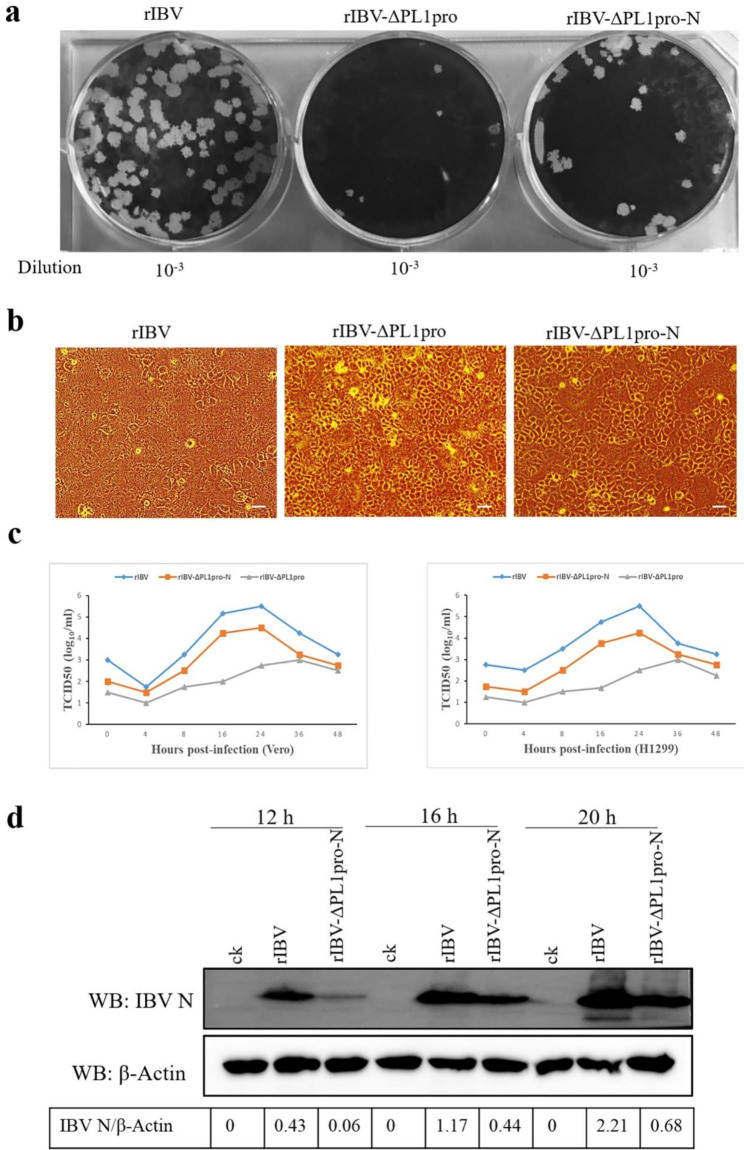
Analysis of the growth properties of rIBV, rIBV-ΔPL1pro, and rIBV-ΔPL1pro-N. (**a**) Plaque formation in rIBV, rIBV-ΔPL1pro, and rIBV-ΔPL1pro-N infected Vero cells. Three viral stock supernatants were serially diluted and inoculated onto the Vero cells for plaque assays, the dilution of the viral stock is indicated. The original images of virus plaque formation are in Additional file 1: Supplemental Fig. 3a. (**b**) CPE formation in rIBV, rIBV-ΔPL1pro, and rIBV-ΔPL1pro-N infected H1299 cells. Scale bars: 50 μm. (**c**) Growth kinetics of the recombinant viruses in Vero cells and H1299 cells. The raw data is presented in Additional file 2. (**d**) Western blot analysis IBV N protein expression. The expression of IBV N protein in H1299 cells infected with rIBV and rIBV-ΔPL1pro-N at a MOI of 0.5 was assessed by Western blot at the indicated times post-infection, respectively, and β-actin was used as an internal control from the same experiment. The full-length blots are in Additional file 1: Supplemental Fig. 3b. Numbers below the image was IBV N/β-actin ratios of band optical density values from Image J software

To examine the potential impact of PL1pro deletion on the genetic stability and development characteristics of the rescued virus, recombinant viruses rIBV and rIBV-ΔPL1pro were cultured on Vero cells for 3 passages, and the plaque size and virus titers were evaluated. In cells infected with rIBV-ΔPL1pro, the average plague size was 0.42 ± 0.17 mm, which was smaller than the average plaque size of 1.25 ± 0.12 mm in cells infected with rIBV (Fig. 3a). The virus titers of rIBV-ΔPL1pro, as calculated by plaque forming units, are significantly lower than those of rIBV (Table 1). In addition to Vero cells, the Human non-small cell lung carcinoma cell line (H1299), which is also permissive to the IBV-Beaudette strain, was used to analyze the growth properties of rescued viruses. Compared to rIBV, the replication of rIBV-ΔPL1pro was also weakened in H1299 cells, as evidenced by the decreased degree of CPE and slower growth (Fig. 3b). Subsequently, we assessed the growth kinetics of rIBV and rIBV-ΔPL1pro in both Vero cells and H1299 cells (Fig. 3c). The titer of rIBV peaked at 24 h post-infection, whereas rIBV-ΔPL1pro reached its peak titer at 36 h post-infection. The titers of rIBV-ΔPL1pro were significantly lower than those of rIBV at various time points during infection. These results indicate that PL1pro significantly influences viral replication in cells and cytopathicity.

Sequence alignment revealed that the amino acid residues in the N-terminal and C-terminal regions of IBV PL1pro are highly conserved. Therefore, one or more of these conserved amino acid residues might be the key factor in determining the reduced pathogenicity of the PL1pro-deleted recombinant virus. To confirm this hypothesis, we constructed the PL1pro-N-deleted recombinant virus rIBV-ΔPL1pro-N and evaluated the growth properties of rIBV and rIBV-ΔPL1pro-N in cells. Similar to rIBV-ΔPL1pro, the replication of rIBV-ΔPL1pro-N was diminished in both Vero and H1299 cells (Fig. 3a-c), displaying smaller plaques (0.74 ± 0.08 mm) and decreased titers (Table 1). Furthermore, the expression of IBV N was detected to quantify the virus replication using Western blot (Fig. 3d). At various infection time points, IBV N protein levels were significantly lower in cells infected with rIBV-ΔPL1pro-N than in those infected with rIBV. The results suggest that the N-terminal region of PL1pro plays a critical role in the diminished replication of PL1pro-deleted recombinant virus.

**Table 1:** Titers of rIBV, rIBV-ΔPL1pro, and rIBV-ΔPL1pro-N

Virus	Virus titer (log10 PFU/ml)	p
rIBV	6.44 ± 0.20	
rIBV-ΔPL1pro	4.58 ± 0.14	0.012*
rIBV-ΔPL1pro-N	5.65 ± 0.04	0.024*

Data were presented as mean ± SD (N = 3). *P < 0.05

### The absence of the N-terminal region of PL1pro renders IBV less resistant to IFN and promotes IFN-β Production during IBV Infection

**Fig. 4:**
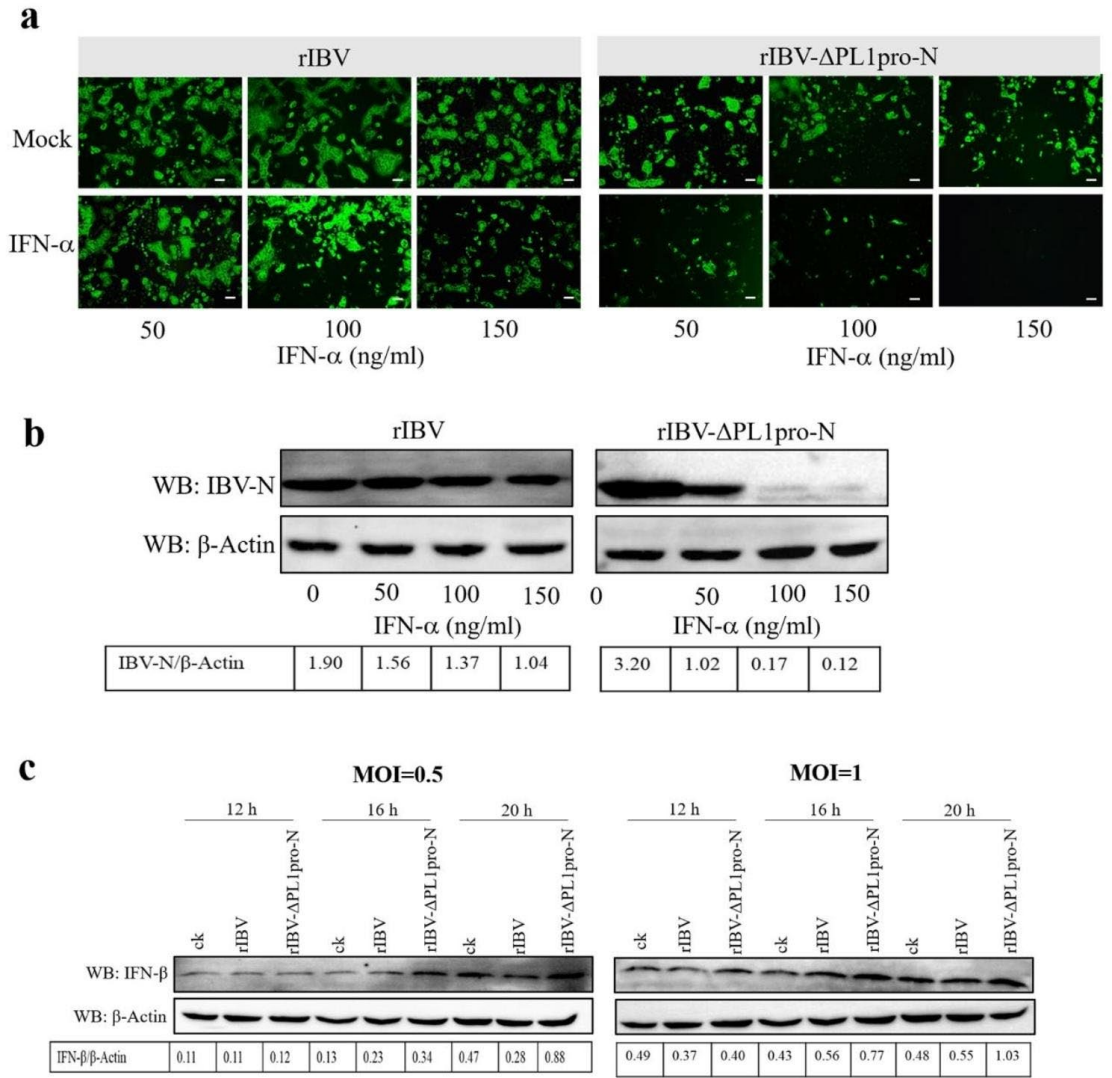
The N-terminal region of PL1pro confers resistance to treatment of IBV with type I IFN and regulates the production of IFN-β. (**a**) Indirect immunofluorescence assay. H1299 cells were prestimulated with the indicated concentrations of IFN-α (50, 100, 150 ng/ml) for 2 h and subsequently infected with rIBV and rIBV-ΔPL1pro-N at an MOI of 0.5. At 18 h post-infection, cells were fixed and stained for IBV-N(green). Scale bars: 50 μm. (**b**) Western blot analysis IBV N protein expression. H1299 cells were prestimulated with IFN-α for 2 h and subsequently infected with rIBV and rIBV-ΔPL1pro-N at an MOI of 0.5. At 18 h post-infection, the expression of IBV N protein was assessed by Western blot. The full-length blots are in Additional file 1: Supplemental Fig. 4a. (**c**) Absence of the N-terminal region of PL1pro upregulates the expression of IFN-β. H1299 cells were infected with rIBV and rIBV-ΔPL1pro-N at MOI of 0.5 and 1, respectively, Western blot was used to detect the expression of IFN-β at the indicated times post-infection, respectively. The full-length blots are in Additional file 1: Supplemental Fig. 4b

Previous studies have shown that coronaviruses use a variety of strategies to counteract the innate immune response, thereby facilitating their replication [19, 20]. To elucidate the underlying mechanisms through which PL1pro affects virus replication, we assessed the IFN response in cells infected with rescued viruses. Given that Vero cells are deficient in IFN [21], we investigated the IFN response in H1299 cells (IFN competent). H1299 cells were pretreated with various concentrations of recombinant human IFN-α then infected with rIBV and rIBV-ΔPL1pro-N. Immunofluorescence staining and Western blot were used to assess the effect of PL1pro N-terminal region on IBV sensitivity to IFN treatment. The results revealed that IFN-α treatment had a minimal effect on virus replication in rIBV-infected cells; only a high IFN-α (150 ng/ml) effectively inhibited rIBV replication. However, in rIBV-ΔPL1pro-N infected cells, even a low concentration of IFN-α (50 ng/ml) significantly hampered virus replication (Fig. 4a and b). Concurrently, Western blot analysis was employed to determine IFN-β expression in rIBV and rIBV-ΔPL1pro-N infected H1299 cells, as shown in Fig. 4c. At 12 h post-infection, no significant difference was observed in the expression level of IFN-β, while at 16 and 20 h post-infection, the expression of IFN-β in rIBV-ΔPL1pro-N infected cells gradually increased compared to that of rIBV. These findings demonstrated that IBV PL1pro may counteract the type I IFN response to support virus replication.

### The absence of the N-terminal region of PL1pro promotes the phosphorylation of STAT1 and nuclear expression of p-STAT1

**Fig. 5:**
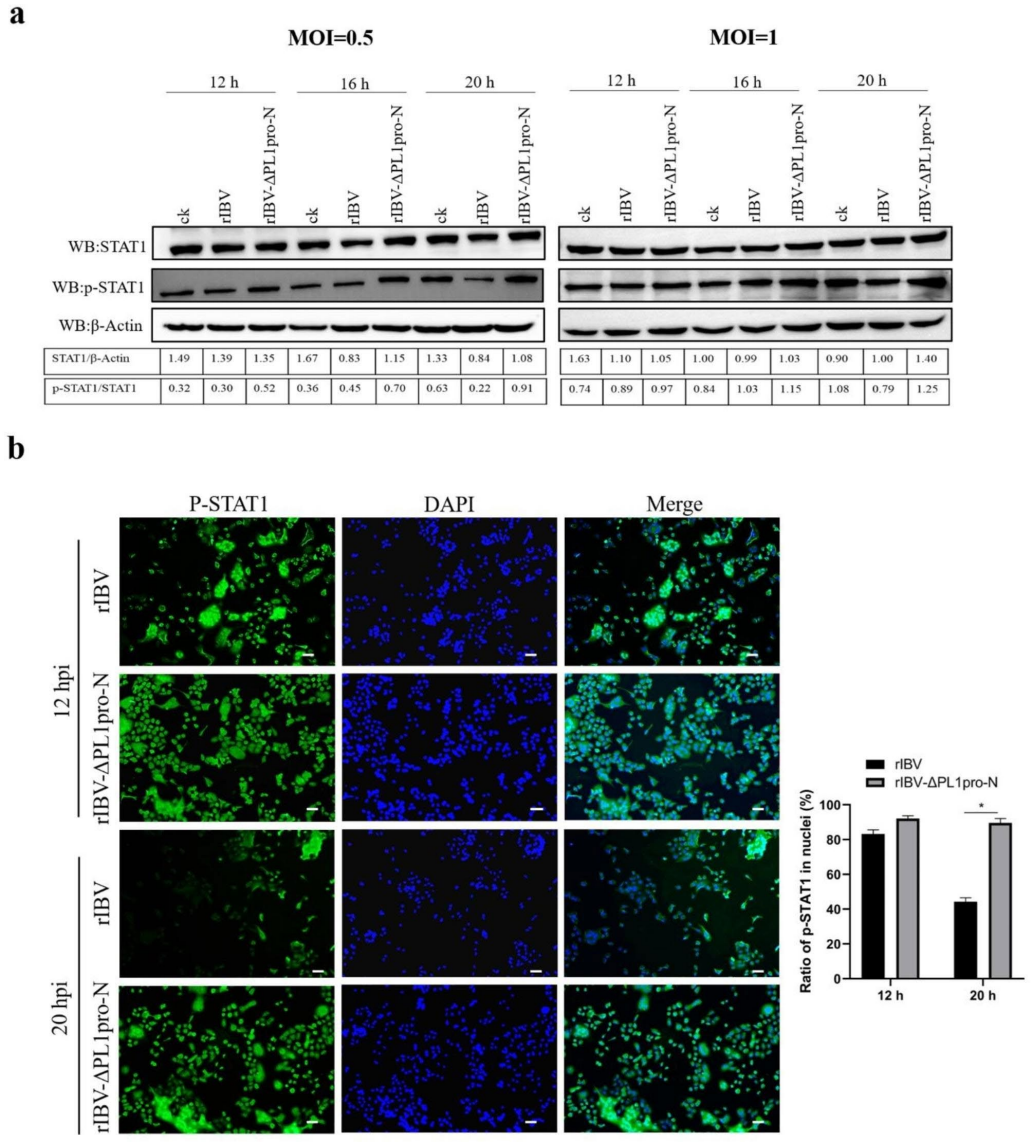
Absence of the N-terminal region of PL1pro promotes phosphorylation of STAT1 and nuclear expression of p-STAT1. (**a**) The absence of the N-terminal region of PL1pro upregulates the expression of p-STAT1. H1299 cells were infected with rIBV and rIBV-ΔPL1pro-N at MOI of 0.5 and 1, respectively, then stimulated with IFN-α (100ng/ml) at 6 h post-infection. Western blot was used to detect the expression of STAT1 and p-STAT1 at the indicated times post-infection, respectively. The full-length blots are in Additional file 1: Supplemental Fig. 5. (**b**) Immunostaining against p-STAT1 in nuclei of H1299 cells. H1299 cells were infected with rIBV and rIBV-ΔPL1pro-N at a MOI of 0.5. At the indicated times post-infection, cells were fixed and stained for p-STAT1 (green). The histogram shows the nuclear expression ratio of p-STAT1. Data were presented as mean ± SD (N = 3). *P < 0.05. Scale bars: 50 μm

Next, we investigate the downstream signaling pathway of IFN to further elucidate the specific mechanism of PL1pro-N affecting virus replication. JAK/STAT1 is the main antiviral signaling pathway of IFN. Type I IFNs activate the JAK/STAT signaling pathway to induce the production of IFN-stimulated genes (ISGs), which inhibit viral replication through various mechanisms [22]. Phosphorylation of STAT1 is a crucial step in activating the JAK/STAT signaling pathway. Therefore, we analyzed the phosphorylation level of STAT1 in IBV-infected H1299 cells using Western blot. While the overall levels of STAT1 exhibited a slight increase at 16 and 20 h post-infection in cells infected with rIBV-ΔPL1pro-N compared to rIBV, the expression of p-STAT1 was notably elevated during these post-infection times in the rIBV-ΔPL1pro-N infected cells, peaking at 20 h post-infection (Fig. 5a). It is well-understood that p-STAT1 can partner with STAT2 and IRF9 to create the interferon-stimulated gene Factor 3 (ISGF3). Upon its translocation to the nucleus, ISGF3 binds to interferon-stimulated response elements (ISREs) within the regulatory region of ISG promoters, initiating the transcription of ISGs [23]. We subsequently examined the nuclear expression of p-STAT1 by immunostaining against p-STAT1, as shown in Fig. 5b. At 12 h post-infection, the nuclear expression levels of p-STAT1 were similar between cells infected with rIBV and rIBV-ΔPL1pro-N. However, at 20 h post-infection, the nuclear expression level of p-STAT1 was significantly higher in rIBV-ΔPL1pro-N infected cells compared to rIBV-infected cells. These findings revealed that the N-terminal region of PL1pro can affect the phosphorylation of STAT1 and nuclear translocation of p-STAT1.

### The absence of the N-terminal region of PL1pro up-regulates the expressions of IFIT family members

**Fig. 6:**
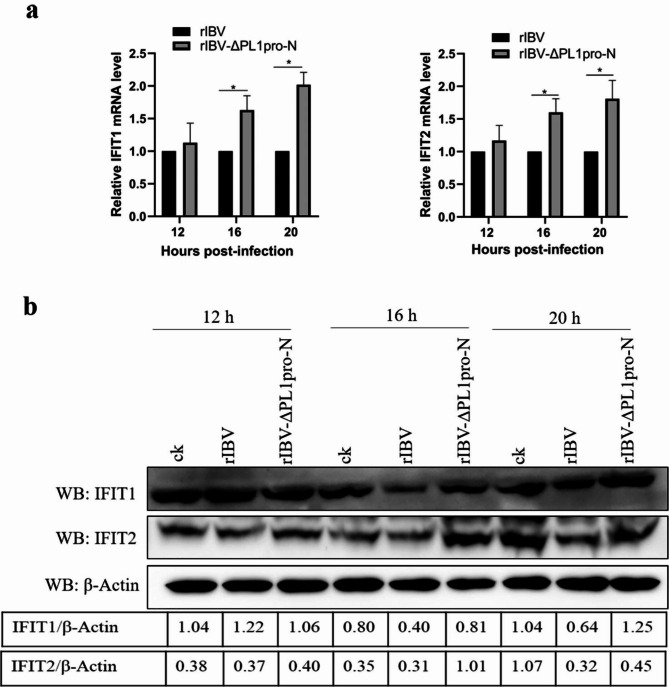
Absence of the N-terminal region of PL1pro up-regulates the expressions of IFIT family members during later stages of infection. (**a**) mRNA expressions of ISGs in rIBV and rIBV-ΔPL1pro-N infected cells. H1299 cells were stimulated with IFN-α (100ng/ml) at 6 h post-infection, and qRT-PCR was performed to analyze the mRNA expressions of IFIT1 and IFIT2. Data were presented as mean ± SD (N = 3). *P < 0.05. The raw data is presented in Additional file 2. (**b**) Protein expressions of ISGs in rIBV and rIBV-ΔPL1pro-N infected cells. H1299 cells were stimulated with IFN-α (100ng/ml) at 6 h post-infection, Western blot was performed to analyze the protein expressions of IFIT1 and IFIT2. The full-length blots are in Additional file 1: Supplemental Fig. 6

The IFIT (interferon-induced proteins with tetratricopeptide repeats) family are prominent ISGs regulated by either viruses or interferons. IFIT proteins have been identified as potent antiviral proteins [24]. To analyze the effect of PL1pro-N on the expression of ISGs, we detected the mRNA and protein expressions of two IFIT family members (ISG56/IFIT1, ISG54/IFIT2) using qRT-PCR and Western blot in rIBV and rIBV-ΔPL1pro-N infected cells. The results demonstrated that both the mRNA and protein levels of IFIT1 and IFIT2 were increased in rIBV-ΔPL1pro-N infected cells at 16 and 20 h post-infection to different degrees (Fig. 6a, b), indicating that the N-terminal region of PL1pro affects the expressions of ISGs during later stages of infection.

### Overexpression of IBV PL1pro-N downregulates the expressions of genes in the IFN pathway

**Fig. 7:**
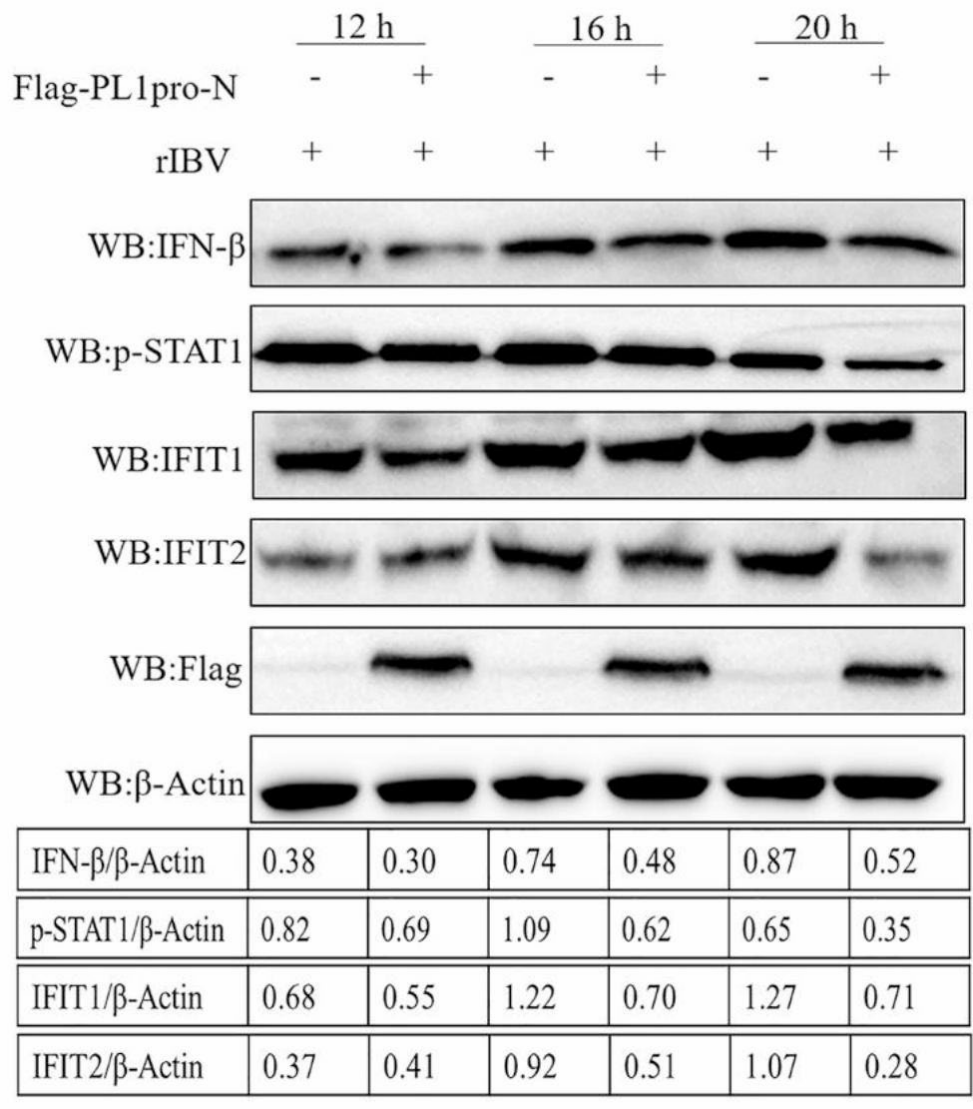
Overexpression of IBV PL1pro-N downregulates the expressions of IFN-β, p-STAT1, IFIT1, and IFIT2 in cells. H1299 cells were overexpressed with IBV PL1pro-N, then infected with rIBV. Western blot was performed to analyze IFN-β, p-STAT1, IFIT1, and IFIT2 protein expressions. The full-length blots are in Additional file 1: Supplemental Fig. 7

To verify the regulatory effect of IBV PL1pro-N on the type I IFN pathway, we constructed plasmid encoding IBV PL1pro-N and overexpressed IBV PL1pro-N in H1299 cells. Western blot was performed to investigate the expressions of IFN-β, p-STAT1, IFIT1, and IFIT2 in cells. As shown in Fig. 7, overexpression of IBV PL1pro-N could downregulate the expressions of genes in the IFN pathway to a different extent at various time points after IBV infection, which further confirm that IBV PL1pro-N has a negative regulatory effect on the innate immune response.

## Discussion

The Beaudette strain of IBV, grown in chicken embryos, was adapted to Vero cells through serial passages, and later this Vero cell-adapted IBV Beaudette strain can also infect human cell lines H1299 and Huh [25]. In our previous study, adaptation of IBV Beaudette strain from chicken embryo to Vero cells showed the accumulation of 49 amino acid mutations. Among them, 26 (53.06%) substitutions were located in the S protein [26]. Suggesting that S gene and its product play important roles in viral adaptation of a new host, mutations in S protein are required for virus to enter the new target cells and spread infection. At present, the results of the current studies on IBV cell adaptability and underlying mechanisms mainly obtained from IBV Beaudette strain. Therefore, we use the non-pathogenic Beaudette strain to investigate the role of PL1pro on IBV replication and the underlying mechanisms.

To our knowledge, this is the first study to elucidate the effect of IBV PL1pro on IBV replication and host innate immune response using a reverse genetic system. The reverse genetic system allows for the production of infectious viruses from cDNAs. This system can be used to adeptly modify viral genomes, facilitating research into the roles of viral proteins in virulence, innate immunity, and both in vitro and in vivo antiviral drug testing [27, 28]. Currently, there are two main reverse genetic systems of IBV. The first system involves inserting a full-length cDNA that matches the IBV genome into the vaccinia virus genome, controlled by the T7 promoter sequence, to produce the recombinant virus [29]. In the second system, the full-length IBV cDNAs are assembled from a contiguous panel of five cDNA fragments that span the entire viral genome by in vitro ligation. Subsequently, the assembled full-length cDNA is transcribed into capped full-length transcripts in vitro to recover infectious viruses after transfection of susceptible cells [30–32]. In our study, we effectively generated infectious viruses, namely rIBV, rIBV-ΔPL1pro, and rIBV-ΔPL1pro-N, with targeted deletions using the second reverse genetics system to study the function of IBV PL1pro on virus virulence and underlying mechanisms. Despite achieving success in IBV recovery, several issues potentially impact the effectiveness of the reverse genetic system. The primary factor influencing the success of a reverse genetic system is the presence of mutations and deletions in the cDNA plasmids. Due to their large size, the IBV cDNA plasmids are susceptible to mutations and deletions when amplified in *E. coli*. To ensure the accuracy of the genomic sequence, we perform sequencing on each IBV cDNA plasmid before initiating virus recovery in our reverse genetic system. Another factor that impacts the success of a reverse genetic system is the lower efficiency of electroporation. During the virus recovery process, we observed variability in the electroporation efficiency of Vero cell lineages. Therefore, enhancing electroporation efficiency and increasing the success rate of reverse genetics are essential challenges that need to be addressed in the future. Addressing these challenges will enable a broader utilization of these techniques for studying viral protein function.

Coronaviruses encode either a single or two PLpros, known as PL1pro and PL2pro, which are significant subdomains of nsp3 [33, 34]. Although many studies have investigated the structures and functions of PLpros, research on IBV PL1pro has been limited due to its proteolytic deficiency. Our initial investigation of IBV PL1pro discovered that although it is not essential for virus replication, it can impact virus replication and pathogenicity. The deletion of PL1pro results in impaired virus replication. Sequence analysis revealed conservation of the N-terminal and C-terminal amino acids, while the amino acids between them exhibited high variability. To identify the pathogenicity region in PL1pro, we recovered the recombinant virus rIBV-ΔPL1pro-N by deleting the N-terminal region of PL1pro. As anticipated, we observed impaired virus replication in the absence of PL1pro-N. Therefore, the N-terminal region of PL1pro plays a crucial role in maintaining the biological function of PL1pro and serves as one of the pathogenic determinants of PL1pro. However, the specific amino acid residues responsible for the reduced virulence of recombinant virus rIBV-ΔPL1pro-N remain unknown. Future studies should identify the key amino acid residues in PL1pro -N that affect viral virulence by obtaining multiple recombinant viruses with point mutations or deletion mutations through IBV reverse genetic system.

The innate immune system, which consists of immune cells, immune tissues, and immune organs, plays a crucial role in the initial detection and restriction of pathogens [35]. However, IBV has developed multiple methods to evade the host’s innate immune response [36]. The structural nucleocapsid (N) protein of IBV can suppress type I IFN production by interfering with the binding of melanoma differentiation-associated gene 5(MDA5)-dsRNA [37]; IBV nsp14 inhibits the JAK/STAT signaling pathway by degrading JAK1 in chicken macrophage cells [38]; The nsp15 of IBV supports virus replication by interfering with the formation of antiviral stress granules (SGs) through antagonizing the activation of PKR and regulating the accumulation of viral dsRNA [39]. In this study, we demonstrated that a proteolytically defective fragment of the PL1pro protein, which IBV codes, plays a significant role in providing resistance to the host’s innate immune response. Knockout of the PL1pro-N decreased IBV resistance to IFN treatment and increased the production of IFN-β in infected H1299 cells, while overexpression of PL1pro-N downregulated the production of IFN-β, suggesting that the effect of PL1pro-N on IBV propagation is related to the regulation of the IFN pathway. Coronavirus PLpros can inhibits IFN production by different strategies. Severe acute respiratory syndrome coronavirus (SARS-CoV) PLpro physically interacts with TRAF3, TBK1, IKKε, STING, and IRF3, disrupt the interaction between the components in STING-TRAF3-TBK1 complex, inhibits STING/TBK1/IKKε-mediated activation of type I IFNs [40]. Severe acute respiratory syndrome coronavirus 2 (SARS-CoV-2) PLpro also targets the STING-IKKε-IRF3 complex to inhibit the production of IFN-β and IFN-stimulated cytokines and chemokines [41]. The IBV PLpro enzyme inhibits the synthesis of IFN-β in infected chicken embryonic fibroblast (DF-1) cells, and this activity is enhanced in the presence of MDA5 and TANK binding kinase 1 (TBK1) [42]. However, additional research is required to identify how exactly the IBV PL1pro-N counteracts the production of type I IFN in the future study.

Previous studies have also shown the resistance of IBV to IFN treatment and further demonstrated that the resistance has been ascribed to the ability of IBV to inhibit the phosphorylation of STAT1 and nuclear translocation of p-STAT1 [43]. Given the observed reduction in resistance of rIBV-ΔPL1pro-N to IFN, we investigated the potential impact of IBV PL1pro-N on the phosphorylation of STAT1 and nuclear translocation of p-STAT1. We observed that knockout of PL1pro-N promoted the phosphorylation of STAT1 at various time points after IBV infection and the nuclear translocation of pSTAT1 at 20 h post-infection. At the same time, the expressions of ISGs (ISG56/IFIT1, ISG54/IFIT2) induced by the IFNs-activated signal transduction cascade were also upregulated in the absence of the PL1pro-N. The results indicate that IBV PL1pro-N is involved in both the induction of type I IFN as well as the IFN-induced antiviral response. Further PL1pro-N overexpression experiments subsequently confirmed the role of PL1pro-N in suppression of host innate immune responses. In addition, our data indicated that the regulatory effect of IBV PL1pro-N on IFN signaling mainly occurs at 16 and 20 h post-infection, while at 12 h post-infection, the regulatory effect is not obvious, suggesting a time-dependent inhibition by IBV PL1pro on host innate immune response. It has been shown that accessory protein ORF 6 of SARS-CoV is involved in blocking nuclear translocation of STAT1 by tethering nuclear import factors on the rough endoplasmic reticulum (ER)/Golgi membrane, inhibiting expression of STAT1-activated genes [44], whether IBV PL1pro-N regulates nuclear translocation of p-STAT1 by the same mechanism needs to be further explored. At the same time, it’s worth noting that, in this study, the absence of PL1pro and the N-terminal region of PL1pro weakened the replication of IBV both in IFN-competent H1299 cells and IFN-deficient Vero cells, suggesting that non-IFN-dependent mechanisms are also involved in the effect of PL1pro on viral replication, further research is necessary to investigate and verify this hypothesis thoroughly.

## Conclusions

**Fig. 8:**
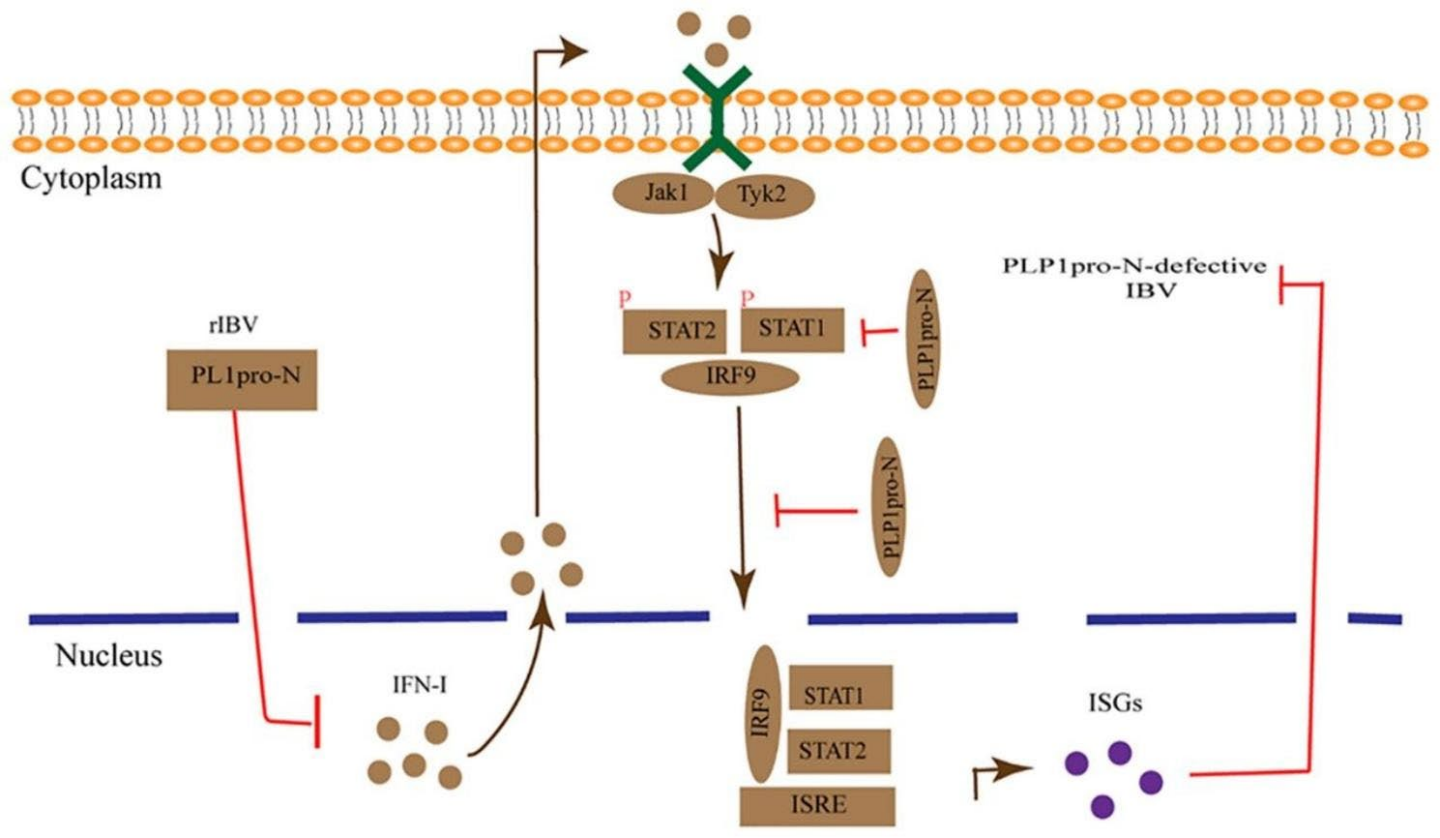
The working model of IBV PL1proN on virus replication and innate immune response

In summary, our study demonstrated the effect of IBV PL1pro on IBV replication and host innate immune response. The results showed that IBV PL1pro and the N-terminal region of PL1pro were involved in virus replication. In the following underlying mechanisms investigation, we found that IBV PL1pro-N was responsible for the observed IFN resistance by IBV. In addition, IBV PL1pro-N regulated IFN production and signaling pathways downstream of IFN (Fig. 8).

## Materials and methods

### Cells, viruses, and plasmids

H1299 cells were purchased from Procell Life Science&Technology Co.,Ltd (China). Vero cells were provided by American Type Culture Collection (ATCC No.: CCL-81). Both cell lines were maintained in Dulbecco’s Modified Eagle Medium (DMEM, Gibco, USA) and supplemented with 10% fetal bovine serum (FBS, Gibco, USA), 100 µg/mL streptomycin and 100 U/mL penicillin (Gibco, USA) at 37^◦^C in 5% CO_2_. IBV Beaudette strain (GenBank accession number: DQ001339) was preserved in our laboratory (Yangtze University, China). The recombinant viruses rIBV, rIBV-ΔPL1pro, and rIBV-ΔPL1pro-N were constructed in this study by reverse genetics, as further detailed below. Plasmids pKTO-IBV-A, pGEM-IBV-B, pXL-IBV-C, pGEM-IBV-D, pGEM-IBV-E bearing IBV Beaudette fragment A, B, C, D, and E covering the full-length genome and plasmid pKTO-IBV-N containing N gene and 3’-UTR were preserved in our laboratory.

### Overexpression and deletion plasmid construction

Plasmid encoding IBV PL1pro-N was generated by amplifying cDNA from pKTO-IBV-A using the corresponding primers and cloning it into pcDNA5-FLAG. Plasmid pKTO-IBV-ΔPL1pro and pKTO-IBV-ΔPL1pro-N were generated by deletion of the PL1pro domain and the N-terminal region of PL1pro on pKTO-IBV-A using PCR. The constructed plasmids were confirmed by sequencing the whole fragments. Table 2 shows the primer sequences.

**Table 2:** Primers for plasmid construction

plasmids	Primers	Sequences (5′ -3 ′)
pcDNA5-FLAG-PL1pro-N	F	CGCGGATCCCTGCCTCTTGATGAAGAT
	R	CCGCTCGAGTTATTCTTGCGCATCAACAG
pKTO-IBV-ΔPL1pro	F	TAAATACCCTCCAGCCACATGTGAGAAA
	R	ATGTGGCTGGAGGGTATTTAATACTTGC
pKTO-IBV-ΔN-PL1pro	F	TAAATACCCTCAACTGTGTCAACAAGAG
	R	GACACAGTTGAGGGTATTTAATACTTGC

### Transfection

H1299 cells were transfected with the overexpression plasmid pcDNA5-FLAG-PL1pro-N using TurboFect Transfection Reagent (R0531, Thermo Fisher Scientific, USA) following the manufacturer’s instructions when they reached 80% confluence in a 6-well plate. After 24 h of transfection, Western blot was used to detect the overexpression effect.

### Recovery of infectious viruses rIBV, rIBV-ΔPL1pro, and rIBV-ΔPL1pro-N

The wild-type recombinant IBV (rIBV) was constructed by reverse genetics, as described previously [18, 31]. Initially, the BsmB I /Bsa I (R0739S/ R3733S, New England Biolabs, USA) digested products of pKTO-IBV-A, pGEM-IBV-B, and the Bsa I digested products of pXL-IBV-C, pGEM-IBV-D, and pGEM-IBV-E were ligated by T4 ligase (E7664, New England Biolabs, USA). Subsequently, the AB and CDE were ligated overnight to obtain the complete cDNA genome of IBV. Next, the full-length cDNA and EcoR I digested pKTO-IBV-N were transcribed in vitro using RiboMAX™ Large Scale RNA Production Systems-T7 (Promega, USA) and supplemented with a cap structure m7G(5’)ppp(5’)G RNA Cap Structure Analog (S1404S, New England Biolabs, USA). Finally, the capped full-length RNA and IBV-N transcripts were subjected to DNaseI treatment and co-transfected into Vero cells using electroporation at 450 v and 50 µF with a BIO-RAD Gene Pulser II electroporator. The transfected Vero cells were incubated overnight in DMEM containing 1% FBS and then in FBS-free DMEM until CPE was formed. The recovered viruses were plaque purified and passaged on Vero cells 3–5 times, and the virus-containing supernatants were collected. In the process of constructing recombinant viruses rIBV-ΔPL1pro and rIBV-ΔPL1pro-N, plasmid pKTO-IBV-A was replaced by plasmids pKTO-IBV-ΔPL1pro and pKTO-IBV-ΔPL1pro-N respectively. The remaining processes are the same as rIBV.

### Viral plaque formation assay and virus titration

Vero cells were seeded into 6-well plates at 5.0 × 10^5^ cells/well one day before infection. Serial dilutions of each viral stock supernatant were prepared and used for inoculation into Vero cells. After incubating for 2 h at 37℃, the inoculum was removed. Cells were subsequently overlaid with 0.4% agar in FBS-free DMEM, incubated at 37℃ for 3–4 days. After incubation, the cells were fixed with 10% formaldehyde for 10 min and stained with a 0.2% crystal violet solution following the removal of agarose. The plaques were counted, size was measured, and virus titers were calculated according to plaque-forming units (PFUs).

### Growth kinetics of the recombinant viruses on vero cells and H1299 cells

Vero and H1299 cells were infected with rIBV, rIBV-ΔPL1pro, and rIBV-ΔPL1pro-N at a MOI of 0.5. The viral stocks were collected at 0, 4, 8, 16, 24, 36 and 48 h.p.i. TCID50 determined the viral titers: the stocks were serially diluted 10-fold with FBS-free DMEM inoculated to 80% confluence of cells in 96 well plates and incubated at 37˚C for 3–4 days. The CPE was observed 3–4 days after infection, and the TCID50 was calculated using Reed and Munch mathematical analysis.

### Interferon sensitivity assay

H1299 cells were seeded in a 6-well plate and allowed to reach 80% confluence. Before infection, they were pretreated with varying concentrations of recombinant human IFN- (50, 100, 150 ng/ml) for 2 h. The cells were infected with recombinant viruses rIBV and rIBV-ΔPL1pro-N at a multiplicity of infection (MOI) of 0.5. At 18 h post-infection, virus replication was detected using indirect immunofluorescence assays targeting the IBV N protein.

### Reverse transcription (RT)-polymerase chain reaction (PCR) and real-time quantitative RT-PCR (RT-qPCR)

RNAs were extracted from H1299 cells using TRIzol reagent (Ambion, USA) according to the manufacturer’s instructions, and RNA concentrations were determined using an OD1000 instrument. The isolated RNA was reverse-transcribed into cDNA using a PrimeScript RT reagent kit with gDNA Eraser (Takara, Dalian, China) following the manufacturer’s instructions; then, cDNA served as a template for RT-PCR and RT-qPCR. The relative RNA levels in each sample were normalized using cellular GAPDH mRNA. The primers for PCR are shown in Table 3.

**Table 3:** Primers for PCR

Gene	Primers	Sequences (5′ -3 ′)	Product size (bp)
IBV subgenomic mRNA 2	F	CTATTACACTAGCCTTGCGCT	244
	R	GTTAACTACCGCATAAGCACC	
IFIT1	F	TCACCAGATAGGGCTTTGCT	160
	R	CACCTCAAATGTGGGCTTT	
IFIT2	F	GCGTGAAGAAGGTGAAGAGG	196
	R	AATTTGGCAATGCAGGTAGG	
GAPDH	F	GTCAAGGCTGAGAACGGGAA	369
	R	AGTGATGGCATGGACTGTGG	

### Western blot

Cell lysis was conducted using RIPA lysis buffer, and cell debris was removed by centrifugation at 12,000 × g for 10 min. The supernatant was mixed with SDS sample loading buffer and subsequently subjected to electrophoresis on 10% or 12% polyacrylamide gels. The separated proteins were transferred onto polyvinylidene fluoride (PVDF) filter membranes (Millipore, USA). The membranes were blocked with a blocking buffer (5% skimmed milk in TBS with 0.1% Tween 20) at room temperature for 2 h and then incubated with primary antibodies overnight at 4˚C following dilution. After incubation, membranes were washed three times with washing buffer (0.1% Tween in TBS) and incubated with an HRP-conjugated goat anti-rabbit IgG secondary antibody at 37℃ for 2 h. Finally, the samples were detected using a chemiluminescence detection kit following the instructions provided by the manufacturer. The antibodies for Western blot were as follows: anti-IBV-N antibody (prepared in Virology Laboratory of Yangtze University, 1:2000), rabbit anti-IFN-β polyclonal antibody (27506-1-AP, Proteintech, Wuhan, China, 1:2000), rabbit anti-STAT1 polyclonal antibody (10144-2-AP, Proteintech, Wuhan, China, 1:2000), rabbit anti-IFIT1 polyclonal antibody (23247-1-AP, Proteintech, Wuhan, China, 1:2000), rabbit anti-IFIT2 polyclonal antibody (12604-1-AP, Proteintech, Wuhan, China, 1:1000), rabbit anti-phospho-STAT1-Y701 antibody (ab30645, Abcam, UK, 1:1000), HRP-conjugated goat anti-rabbit IgG (Sangon Biotech, Shanghai, China, 1:5000).

### Indirect immunofluorescence assays (IFA)

H1299 cells were cultured in a 24-well plate until reaching 80% confluence and then infected with rIBV and rIBV-ΔPL1pro-N at an MOI of 0.5. Cell culture was discarded at 24 h post-infection. Subsequently, the cells were fixed with 4% paraformaldehyde for 15 minutes, followed by permeabilization with 0.5% Triton-X100 for 15 minutes at room temperature after being washed three times with PBS. The plate was blocked using 5% FBS at room temperature for 1 h, then incubated with anti-IBV-N antibody (1:200) and rabbit anti-phospho-STAT1-Y701 antibody (1:100) at 4 ºC overnight. After washing, the tissue samples were incubated with FITC-conjugated goat anti-rabbit IgG (AS011, ABclonal, Wuhan, China) at room temperature for 1 hour. Nuclei were stained with 4’, 6-diamino-2-phenylindole (DAPI) (RM02978, ABclonal, Wuhan, China) for 15 min. Finally, the images were captured using a fluorescence microscope (Leica DM i8 manual).

### Statistical analysis

Statistical analysis was conducted using SPSS 23.0, while graphs were created using GraphPad Prism 8. The intensity of protein bands was quantified using ImageJ software. The data were represented as mean ± standard deviation (SD) of three independent experiments. The significance between groups was determined using the student’s t-test. P values < 0.05 were considered statistically significant.

### Electronic supplementary material

Below is the link to the electronic supplementary material.

Supplementary Material 1

## Data Availability

The datasets used during the current study are available from the corresponding author on reasonable request.

## Abbreviations

Plpros: Papain-like proteases
IBV: Infectious bronchitis virus
IFN: Interferon
3CLpro: 3 C-like cysteine protease
DUB: Deubiquitinase
TGEV: Transmissible gastroenteritis virus
PEDV: Porcine epidemic diarrhea virus
PCBP2: Poly(C) binding protein 2
CPE: cytopathic effect
ISGs: IFN-stimulated genes
ISGF3: interferon-stimulated gene Factor 3
IFIT: Interferon-induced proteins with tetratricopeptide repeats
MDA5: Melanoma differentiation-associated gene 5
SGs: Stress granules
TBK1: TANK binding kinase 1
DMEM: Dulbecco’s Modified Eagle Medium
FBS: Fetal bovine serum
RT: Reverse transcription
PCR: Polymerase chain reaction
PVDF: Polyvinylidene fluoride
IFA: Indirect Immunofluorescence Assays
SARS-CoV: Severe acute respiratory syndrome coronavirus
SARS-CoV-2: Severe acute respiratory syndrome coronavirus 2
