# Supplementary material for: The role of IBV PL1pro in virus replication and suppression of host innate immune responses
Source: BMC Vet Res. 2023 Dec 13;19:270. doi: 10.1186/s12917-023-03839-2 (PMC10717896; doi:10.1186/s12917-023-03839-2)
Supplement: Supplementary file 1 — Supplementary Material 1 [file 12917_2023_3839_MOESM1_ESM.docx]

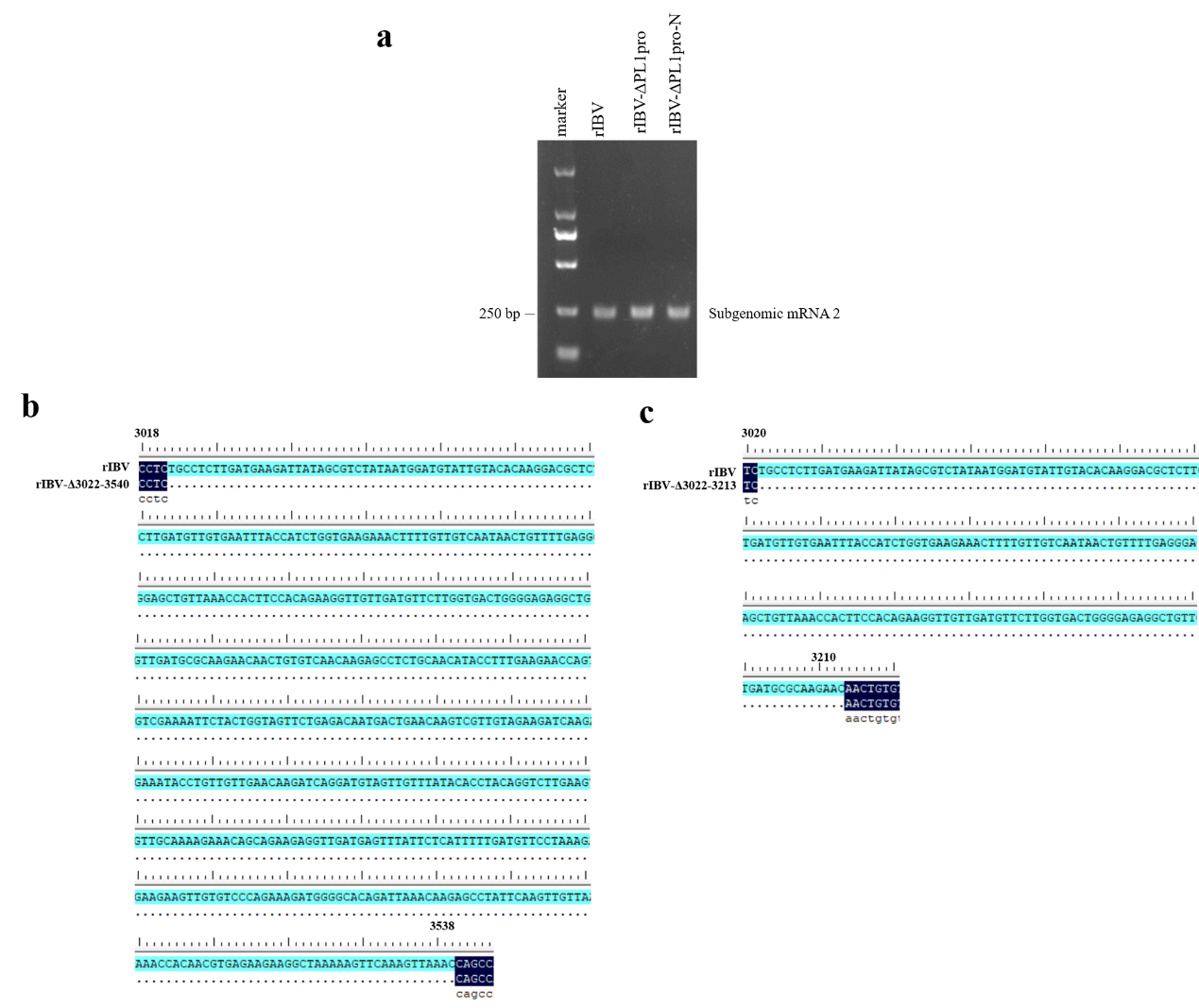


**Supplemental Fig. 1**. Subgenomic mRNA 2 detection and fragment deletion verification of recovered viruses. (a) Subgenomic mRNA 2 detection of rIBV, rIBV-ΔPL1pro and rIBV-ΔPL1pro-N. (b) Fragment deletion verification of rIBV-ΔPL1pro. (c) Fragment deletion verification of rIBV-ΔPL1pro-N.


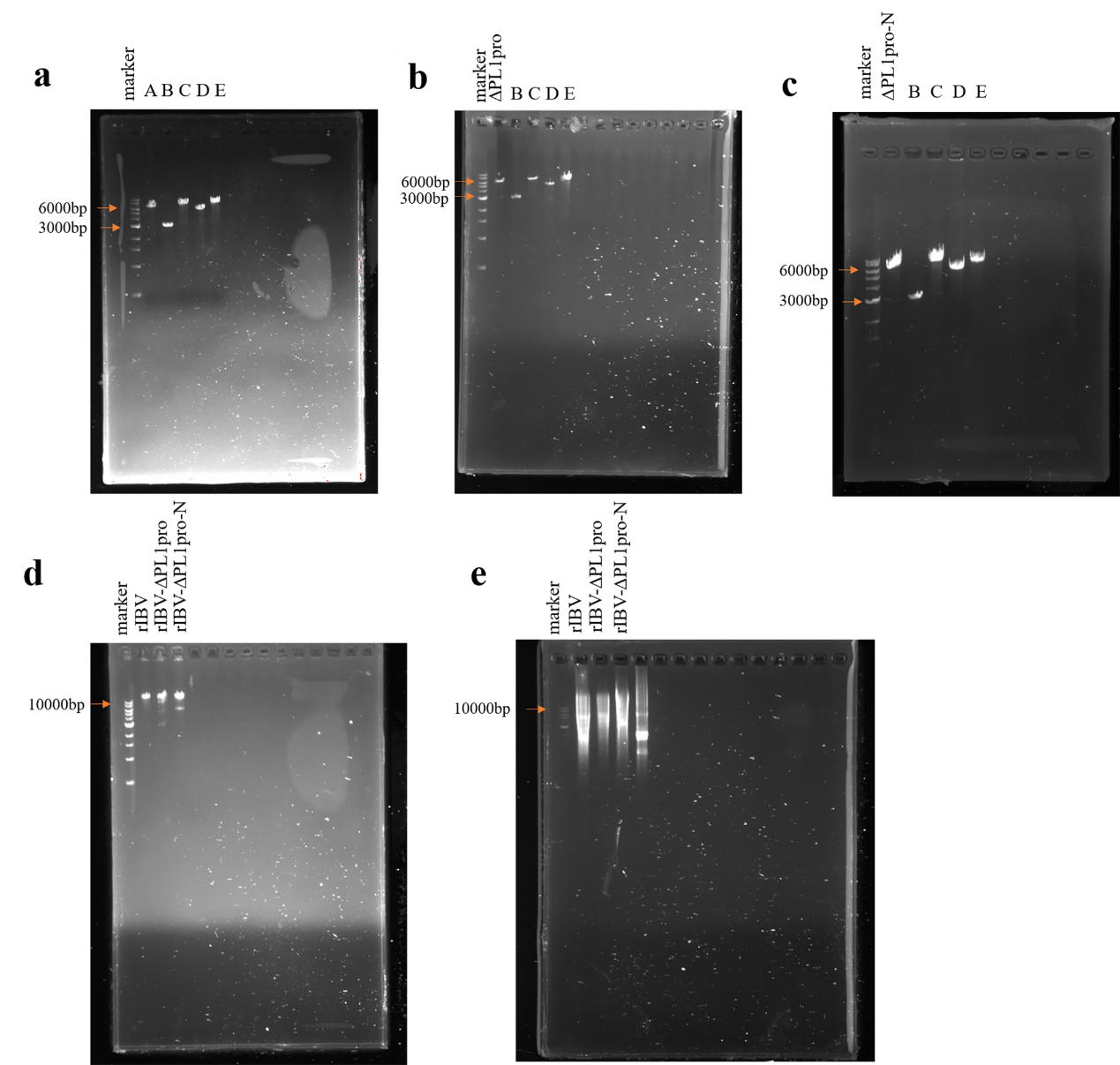


**Supplemental Fig. 2**. In vitro transcription of the full-length transcripts of rIBV, rIBV-ΔPL1pro, and rIBV-ΔPL1pro-N. (a-e) Original gel images in the Fig. 2b.


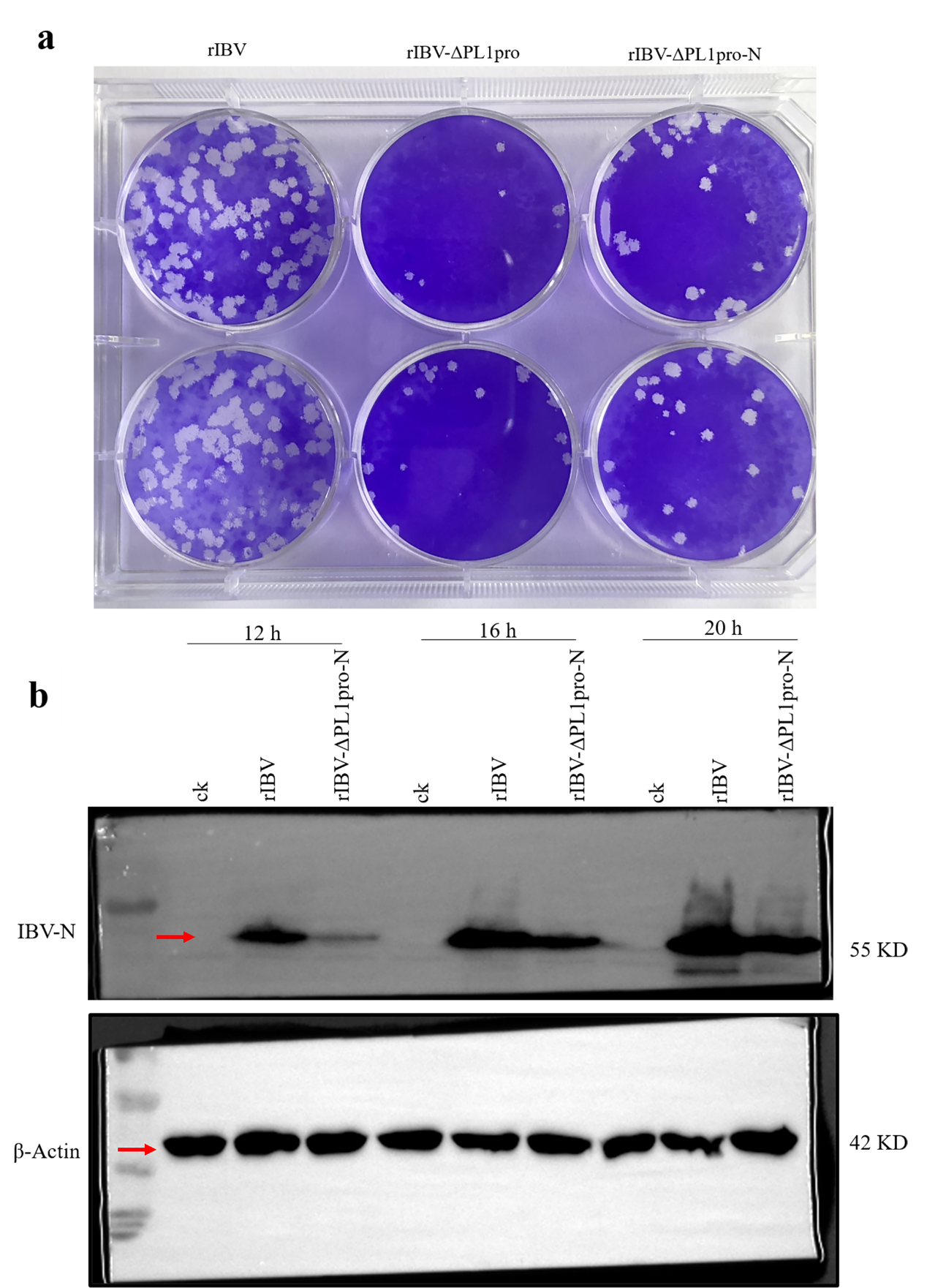


**Supplemental Fig. 3.** (a) The original images of virus plaque formation in the Fig. 3a.

(b) The expression of the IBV N and β-actin proteins were detected by Western blot. Original protein blot images of IBV N and β-Actin in the Fig. 3d


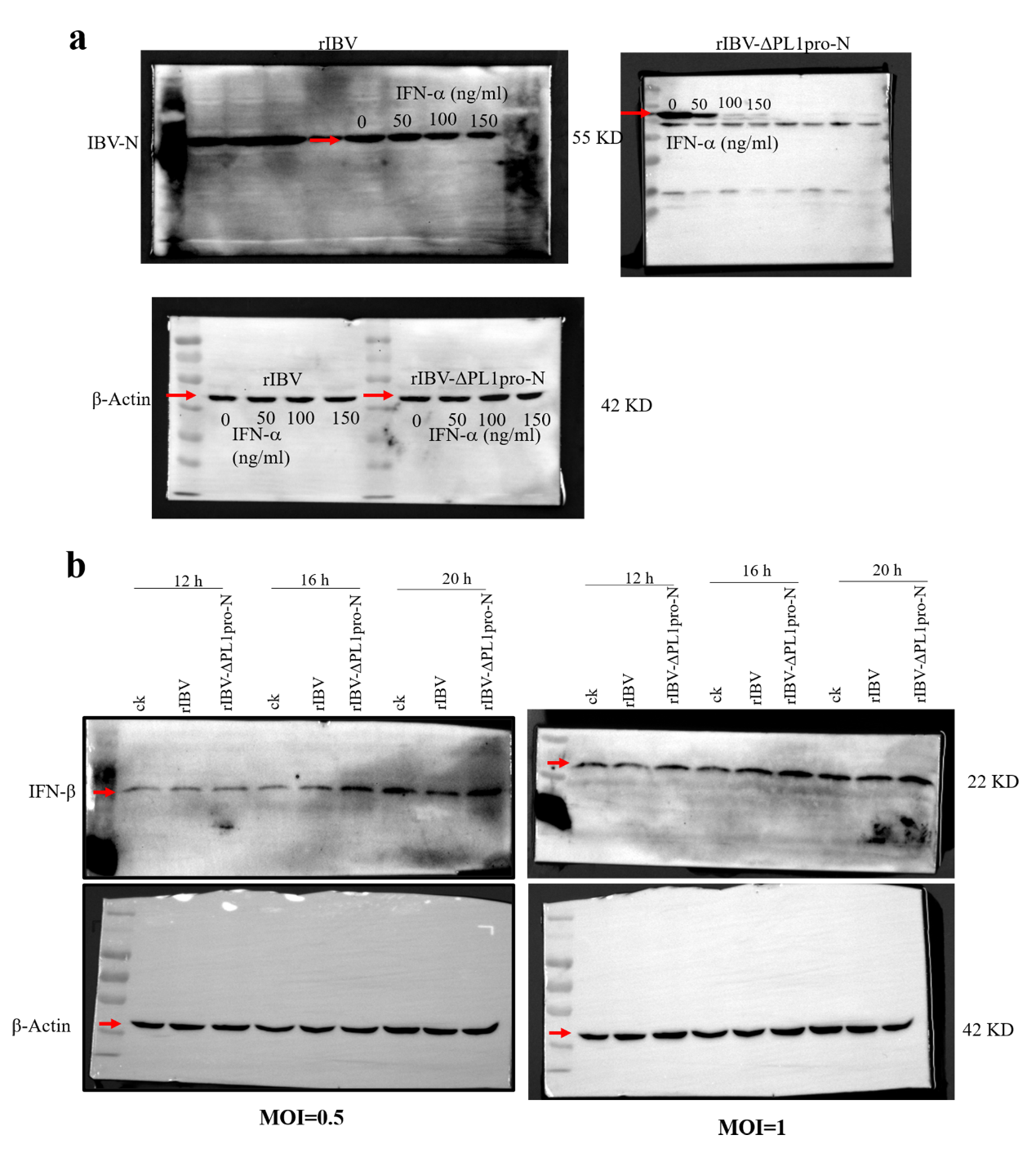


**Supplemental Fig. 4.** (a) The expression of the IBV N and β-actin proteins were detected by Western blot. Original protein blot images of IBV N and β-Actin in the Fig. 4b. (b)The expression of the IFN-β and β-actin proteins were detected by Western blot. Original protein blot images of IFN-β and β-Actin in the Fig. 4c.


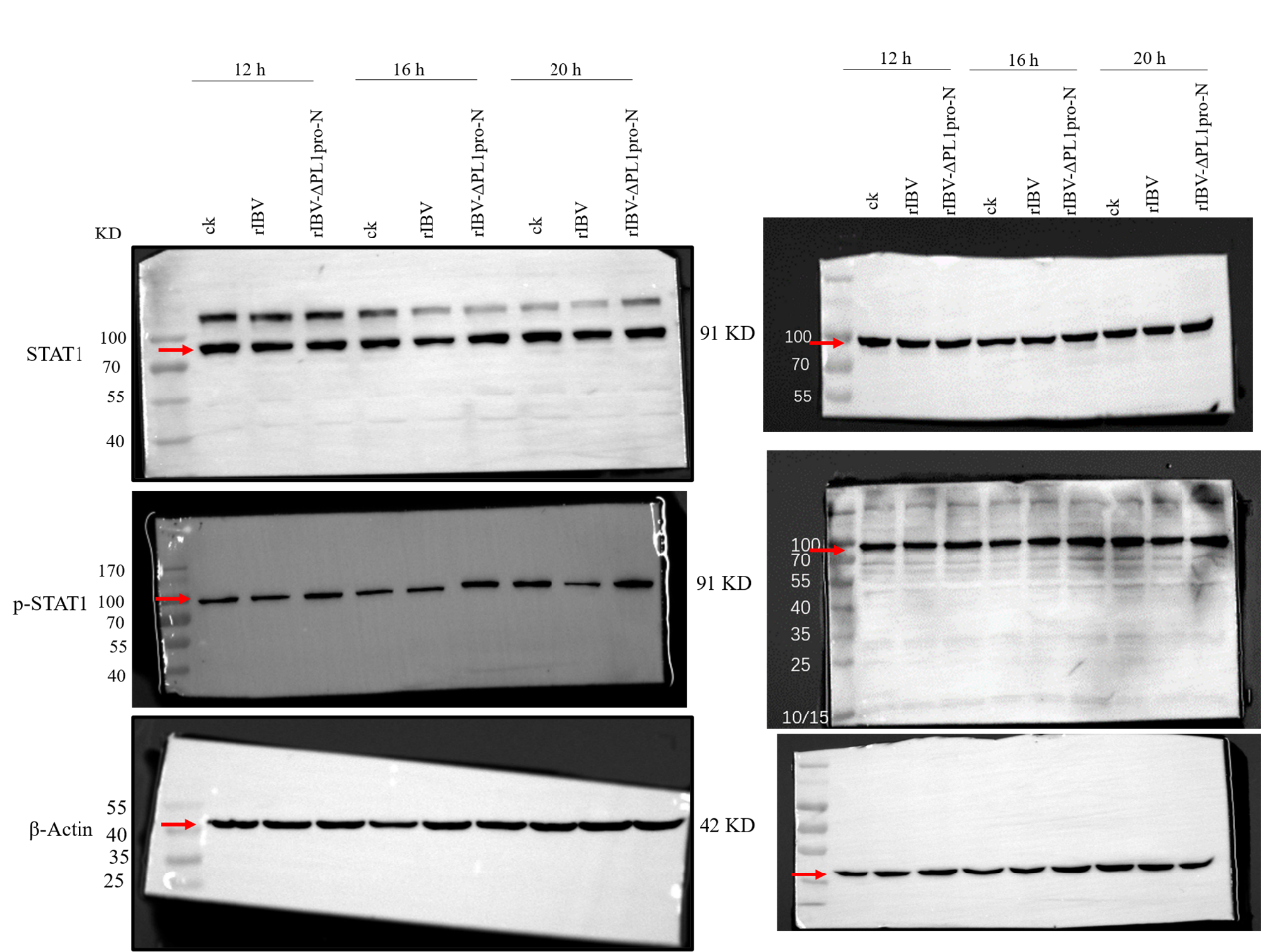


**Supplemental Fig. 5.** The expression of theSTAT1, p-STAT1 and β-actin proteins were detected by Western blot. Original protein blot images of STAT1, p-STAT1 and β-Actin in the Fig. 5a.


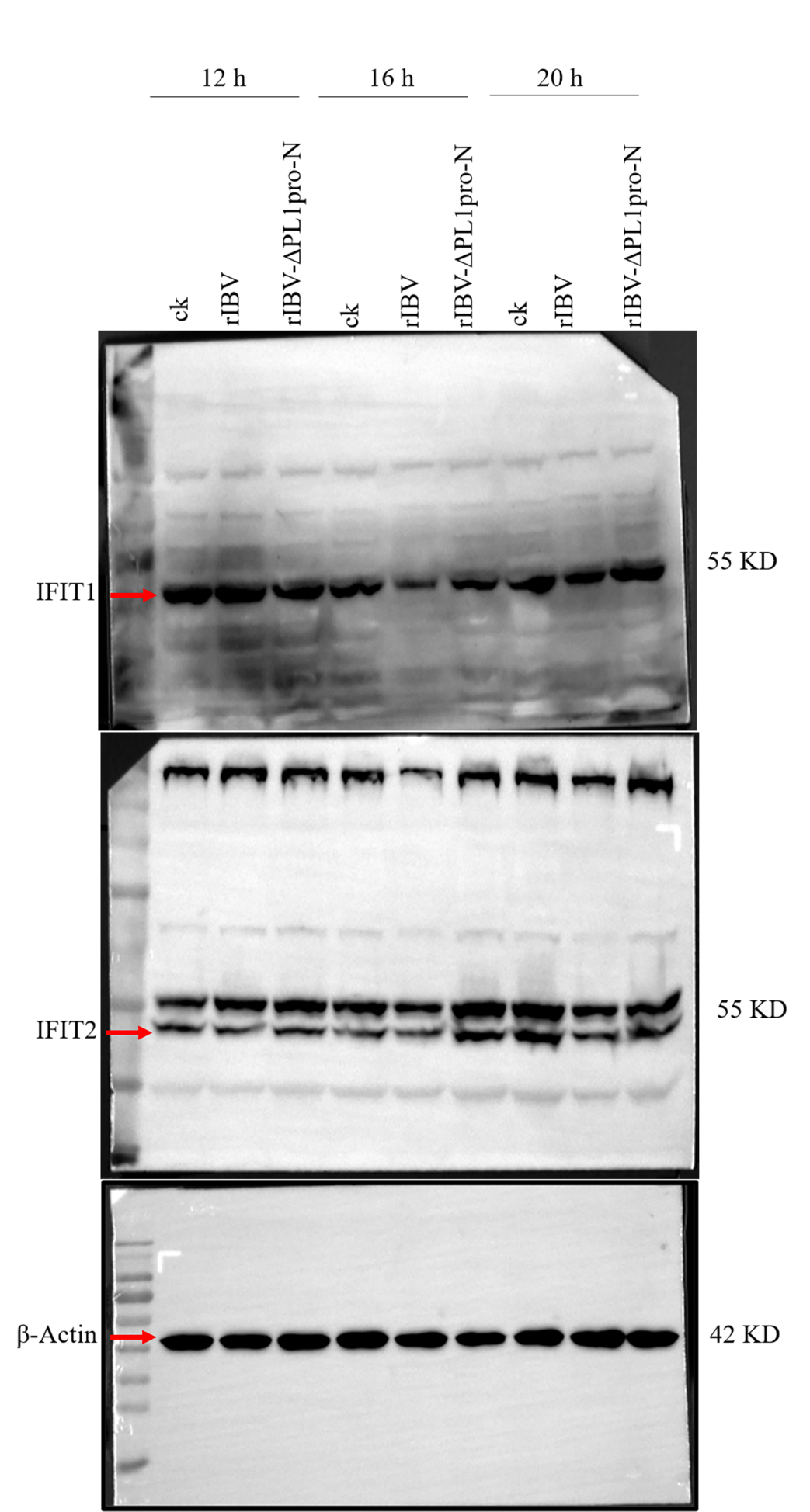


**Supplemental Fig. 6.** The expression of the IFIT1, IFIT2 and β-actin proteins were detected by Western blot. Original protein blot images of IFIT1, IFIT2 and β-Actin in the Fig. 6b.


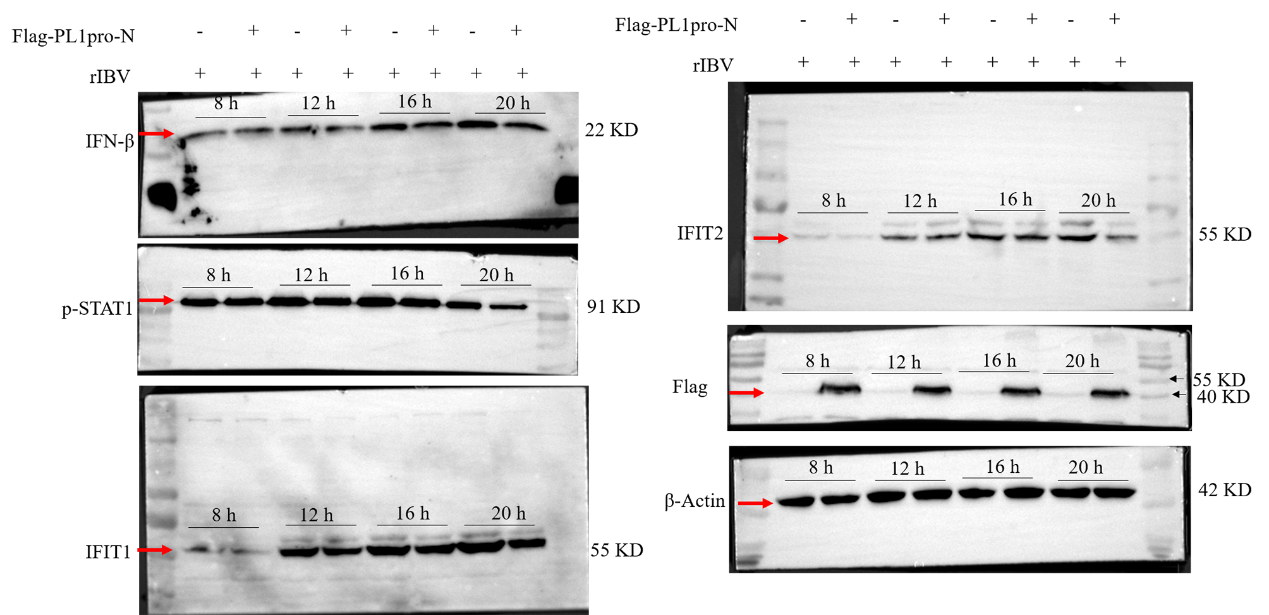


**Supplemental Fig. 7.** The expression of the IFN-β, p-STAT1, IFIT1, IFIT2 and β-Actin proteins were detected by Western blot. Original blot images of IFN-β, p-STAT1, IFIT1, IFIT2 and β-Actin in the Fig. 7.
